# Domain-agnostic weakly supervised surgical instrument segmentation

**DOI:** 10.1038/s41598-026-43054-1

**Published:** 2026-03-18

**Authors:** Rebekka Peter, Doan Xuan Viet Pham, Philipp Matten, Erik Wu, Jonas Nienhaus, Felix Meissen, Martin J. Menten, Eleonora Tagliabue, Franziska Mathis-Ullrich

**Affiliations:** 1 Zeiss Innovation Hub @ KIT, Eggenstein-Leopoldshafen, Germany; 2 Department of Artificial Intelligence in Biomedical Engineering, Friedrich-Alexander-University Erlangen-Nuremberg, Erlangen, Germany; 3 Chair for AI in Healthcare and Medicine, Technical University of Munich, Munich, Germany; 4 Center for Medical Physics and Biomedical Engineering, Medical University of Vienna, Vienna, Austria; 5 BioMedIA, Imperial College London, London, UK

**Keywords:** Biomedical engineering, Endoscopy, Tomography

## Abstract

Recent advancements in visual foundation models open new avenues in the field of surgical instrument segmentation in medical images. Segmentation foundation models provide high segmentation accuracy for objects of interest that are selected via prompts in the form of points, bounding boxes, or text. However, the choice of suitable prompts either requires manual interaction or relies on two-stage pipelines based on supervised, typically domain-specific models. This limits their applicability for domain-agnostic surgical instrument segmentation. We propose a method for surgical instrument segmentation that leverages the power of the segmentation foundation model SAM2 while eliminating the need for a user-defined input prompt or domain-specific annotated datasets. We achieve this by utilizing an anomaly detector generated from non-instrument images to identify instruments as unseen regions and in this way, define a SAM2 input prompt based solely on image-level annotations. For three datasets for surgical instrument segmentation from diverse domains (EndoVis2017, CaDIS, and PASO-SIS), we achieve mean Normalized Surface Distances ranging from ${53}{\%}\,\hbox {to}\,{73}{\%}$. This demonstrates the competitiveness of our method compared to alternatives, while its training- and mask-free nature makes it well-suited for surgical workflow integration. By simplifying surgical instrument segmentation, we advance the field of computer-assisted surgery and unlock a wide variety of assistance functions with minimal effort.

**Supplementary Information:**

The online version contains supplementary material available at 10.1038/s41598-026-43054-1.

## Introduction

Surgical instrument segmentation (SIS) is fundamental for computer-assisted and robotic surgery as it is an essential part for scene understanding. SIS enables downstream tasks like automated alignment of imaging sensors to the surgical instrument^1^. Another example is the masking of surgical instrument regions to exclude them during 4D scene reconstruction^2,3^. The emergence of SIS challenges like the *2017 Robotic Instrument Segmentation Challenge* ^4^ or *CaDIS: Cataract Dataset for Image Segmentation* ^5^ further highlights the interest in this task.

In the last decade, supervised deep learning (DL) has emerged as the most established method for SIS^6^. State-of-the-art deep learning frameworks based on convolutional neural network (CNN) architectures have achieved high accuracy and fast inference times, even in challenging scenes with light reflections^7^, low contrast^8,9^, partial occlusion^10,11^, and small instrument sizes^5^. Despite their effectiveness, current approaches for SIS face certain constraints. Supervised DL methods require large data sets with mask annotations, which acquisition is time consuming, often requires medical expertise, and has to consider patient data privacy issues. Furthermore, supervised models may experience a drop in performance when there is a domain shift between the training and target data^12^.

The recent introduction of Large Vision Models (LVMs) have reshaped the field of object detection and instance segmentation, and the combination of LVMs and large language models (LLMs) provides powerful possibilities. For example, Grounding DINO^13^ and Grounded SAM^14^ combine semantic text encoding with instance detection and segmentation capabilities to solve semantic object detection and semantic segmentation tasks, respectively.

**Fig. 1:**
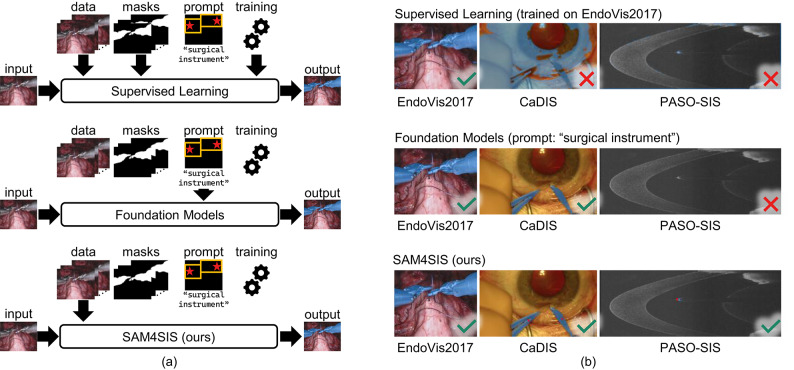
**a** Comparison of requirements for supervised learning and foundation models in SIS versus our proposed approach, which eliminates the need for mask annotations, model training, and user-defined prompts. **b** Demonstration of domain agnosticism. The blue overlays indicate the SIS prediction masks. Supervised models are limited to specific datasets and fail to adapt to shifts in surgical domains or imaging modalities. Foundation models generally struggle to recognize surgical instruments in domains where visual representation differs from natural forms, such as surgical instruments in OCT B-scans. In contrast, our method is domain-agnostic, effectively handling diverse datasets, including EndoVis2017, CaDIS, and PASO-SIS.

When considering foundation models for SIS, following constraints need to be considered: While domain-unspecific foundation models offer a greater ability to generalize, their performance might degrade when applied to medical images as they are trained on natural images^15^. This domain shift is particularly prominent for cross-sectional data representations, intensity-based, and false-color data types where the visual appearance of concepts changes drastically compared to real-world training data. For instance, in EndoVis2017 and CaDIS camera images, surgical instruments can be described as gray, metallic, elongated objects with shafts and various instrument tip shapes. However, in cross-sectional OCT B-scans, these instruments appear as bright crescents or lines, as illustrated in Fig. 1. Ma et al.^15^ demonstrate the superiority of a foundation model trained on multi-modal medical imaging segmentation datasets compared to both a general foundation model and task-specific models. Retraining and fine-tuning of LVMs, particularly for surgical scenes, lead to the bottleneck of necessitating extensive annotations and could potentially diminish their generalization capability^16,17^. Established segmentation foundation models like Segment Anything Model (SAM)^18^and Segment Anything Model 2 (SAM2)^19^ focus on object segmentation, and they lack the ability of prompt-free semantic class assignment^20^. Therefore, the semantic meaning of surgical instruments must be incorporated through input prompts or post-processing techniques which are typically less domain-agnostic and / or are based on supervised learning.

Figure 1 summarizes the required resources of existing methods and categorizes our approach for surgical instrument segmentation. Both supervised learning and foundation models are unsuited for SIS in surgical domains with limited resources for annotated datasets, network training, or manual image-wise prompt selection. In contrast, we propose a two-stage SIS pipeline that does not rely on annotation masks, prompt engineering or supervised (re-)training of segmentation models. We follow the principle of *segmenting anything new*: to localize surgical instruments, we leverage the concept of the feature-based anomaly detector PatchCore^21^. By constructing an encoding space (memory bank) of normal anatomy using data with no surgical instrument present, we are able to identify surgical instruments as unseen objects based on the distance of their features in the encoding space. In doing so, the set of surgical instruments does not need to be defined a priori as surgical instruments are recognized as unseen objects based on high feature distances. From the coarse PatchCore instrument localization, an input prompt for the LVM SAM2^19^ is generated. SAM2 produces an accurate pixel-wise segmentation mask for this prompt, i. e. for surgical instruments.

We evaluate our approach on three challenging and diverse datasets in the field of endoscopic and ophthalmic surgery. First, the publicly available EndoVis2017 dataset^4^ comprises videos captured during abdominal porcine procedures using da Vinci Xi systems^22^. The second dataset, CaDIS^5^, is a publicly available collection of intra-operative microscopic images of the anterior eye during cataract surgery. Thirdly, we utilize our publicly available dataset PASO-SIS^23,24^ of ex-vivo porcine eye anterior segment optical coherence tomography (OCT) scans during tissue manipulation procedures featuring SIS masks. By selecting datasets from different surgical domains and sensor modalities, we demonstrate that our approach is data agnostic. In particular, evaluating OCT data, which differ greatly from typical surgical scenes, demonstrates our method’s robustness to changes in instrument appearance without relying solely on conventional model representations.

Our contributions include:

- We propose a SIS method that relies solely on image-level annotations by treating the localization task as anomaly detection task, alleviating the need for segmentation mask annotations.
- We overcome the need for a user-defined input prompt when utilizing the segmentation capabilities of SAM2.
- We introduce a surgical instrument scoring mechanism (SI filter) that filters out false-positive detections and improves the accuracy of the PatchCore-based localization. Furthermore, we replace the SAM2 internal multi-mask scoring system with our own mechanism (SAM4SIS score) to select the most accurate mask and mitigate ambiguities.
- We demonstrate that our approach is data agnostic and robust to instrument appearances, by evaluating it on datasets from three different surgical domains and imaging modalities.

## Related work

### Weakly supervised learning for surgical instrument segmentation

Due to the high annotation effort required for classical supervised learning approaches in SIS, the interest in developing weakly supervised learning strategies that rely on simple and rapidly generated annotations is high.

One prominent avenue for weakly supervised learning in SIS is the use of simulated image data^25–28^ or synthesized image data generated through image-to-image translation using generative adversarial networks (GANs)^29,30^. The main challenge is keeping the domain gap between the synthetic and real-world images small while providing a high variance in the data. The generation of image simulators and synthesis methods may still require significant effort. Additionally, these approaches may not be agnostic to new domains or surgical instruments, necessitating the creation of new simulations and synthesized data to achieve generalizability across different surgical scenarios. In contrast, we utilize image-level annotations indicating the presence or absence of surgical instruments, which are easily generated. We further do not require descriptive, simulated, or synthetic surgical images.

### Foundation models for surgical instrument segmentation

Zhou et al.^31^ utilize the pretrained vision-language model CLIP^32^ to encode text and images for SIS. Their framework allows incorporating visual characteristics of surgical instruments in text form to enhance segmentation accuracy. Similarly, Soberanis-Mukul et al.^33^ utilize grounded SAM (GSAM) to detect and segment surgical instrument regions based on text inputs. In addition, they utilize the video object segmentation model Cutie^34^ to track and segment objects defined by the initial frame annotations provided with GSAM. Both frameworks aim to speed up the data annotation task by leveraging text prompts. However, the success rates heavily depend on the chosen text prompt and in general, there is no reliable text prompt that can segment surgical instruments effectively across a complete dataset or even across different datasets. Thus, manual prompt selection is needed. As an alternative, Zhaksylyk et al.^35^ discuss bounding-box and point prompts for semi-automated SIS. While bounding-box prompts can be ambiguous when multiple instruments are closely positioned, point prompts offer a more reliable and user-friendly option. However, Zhaksylyk et al. highlight a limitation of point prompts: even slight adjustments in point location can greatly impact the model’s output. They propose a shift block module for SAM2 to enhance robustness, ensuring more consistent and higher-performing results in point prompt-guided SIS.

Attempts to circumvent the bottleneck of explicit user input selection are addressed by Yue et al.^36^ and Sheng et. al.^37^. Yue et al.^36^ efficiently fine-tune the SAM decoder in combination with a prototype-based class prompt encoder. Sheng et al.^37^ utilize the Detection Transformer model DETR^38^ to generate a prompt input for Segment Anything Model (SAM). However, both platforms require a set of annotated data of the target domain and DL training.

### Surgical instrument segmentation for EndoVis2017

The EndoVis2017 challenge^4^ featured 10 teams competing in binary, parts, and type-based segmentation of articulated da Vinci robotic instruments. Our contribution addresses the binary instrument segmentation challenge. The team of MIT^39^ achieved the highest average Intersection over Union (IoU) score of 85.4% in binary segmentation, utilizing *TernausNet*, a U-Net-like architecture with pre-trained encoders. Further optimizations of the U-Net architecture for SIS are summarized by Yang et al.^40^, including *UNet++*^41^ with redesigned skip pathways, *Attention U-Net*^42^ featuring attention gates, *U-NetPlus*^43^ with a pre-trained encoder and a redesigned decoder utilizing nearest-neighbor interpolation instead of transposed convolution, and a U-Net variant by Yang et al.^40^ incorporating a dual-attention module, residual path, and non-local module. Surpassing the challenge winners Shvets et al.^39^, these innovations achieve an IoU of up to 92.74% (EndoVis2017 sequence 1-8).

Focusing on semi-supervision, Wei et al.^44^ introduced *SegMatch*, adapting the FixMatch semi-supervised classification pipeline for semi-supervised segmentation by combining consistency regularization and pseudo-labeling. Using 30% (1585) of available segmentation masks from EndoVis2017, SegMatch achieved a mDICE of 89.2%, outperforming other semi-supervised and supervised methods on the EndoVis2017 dataset. When reducing the number of used segmentation masks to 10% (528), the performance decreases to a mDICE of 81.1%, which indicates the reliance on a minimum amount of mask annotations.

Sestini et al.^45^ proposed *FUN-SIS*, a fully unsupervised approach leveraging noisy pseudo labels from motion information and shape priors from existing SIS datasets, reducing the need for manual annotations. While being effective for EndoVis2017 with a mDICE of 76.25%, this method may struggle with custom data where instrument shapes are not well-represented in existing datasets, such as OCT scans. Liu et al.^46^ achieve a mean Intersection over Union (mIoU) of 72.07% by leveraging temporal video data and motion information, effectively eliminating the reliance on surgical shape priors.

In the category of training-free, semi-automated SIS, Ying et al.^47^ extend the SAM2 tracker with novel context-aware and occlusion-resilient memory modules, achieving a mIoU of 62.49% for multi-instrument tracking. While outperforming the vanilla SAM2 tracker, the need for manual input prompts during initialization remains a bottleneck for automation.

### Surgical instrument segmentation for CaDIS

Grammatikopoulou et al.^5^ benchmark the performance of state-of-the-art deep learning models for semantic segmentation on the CaDIS dataset. On the task of pixel-wise segmentation for eight classes, the highest performance was achieved with *HRNetV2*^48^ with a mIoU of 84.9%. With an mIoU of 77% vs. 85% for the instrument vs. anatomy classes, respectively, the segmentation of surgical instruments proved to be particularly challenging. This can be attributed to several factors such as class imbalance, the small size of surgical instruments, and high variability in their appearance.

Ni et al.^49^ evaluate the performance of various state-of-the-art supervised networks on CataIS, a dataset similar to CaDIS with eleven surgical instrument classes. They report an IoU ranging from 56.00% with U-Net to 89.14% with their own model, *SurgiNet*, which is optimized for changes in lighting conditions present in CataIS.

### Surgical instrument segmentation for ophthalmic OCT

Weiss et al.^2^ and Heo et al.^50^ investigated SIS in axial projections of OCT volumes and B-scans, respectively. In the former study, a real-time rendering pipeline was developed that decomposed the scene into a static and dynamic part, allowing for the alignment and temporal fusion of the static parts to improve image quality. For each volume, four variants of 2D axial projections were generated, and a lightweight segmentation network based on U-Net^51^ and ResNet-18^52^ was trained using approximately 480 samples to achieve an instrument class accuracy of 85.33%. In the latter study, U-Net was utilized as well and trained on 244 data samples, achieving an mIoU of 66.4% and 62.6% for the forceps and needle classes, respectively.

Nienhaus et al.^23^ present U-Net as a supervised learning baseline for binary segmentation in porcine eye anterior segment OCT scans. Utilizing 76% (775) of the annotated data for training, they achieve a mIoU of 57.72%. This result underscores the challenges posed by the domain’s characteristics, such as significant class imbalance with instruments occupying only 0.18% of the B-scans, and unavoidable inter-annotation variance.

## Methods

**Fig. 2:**
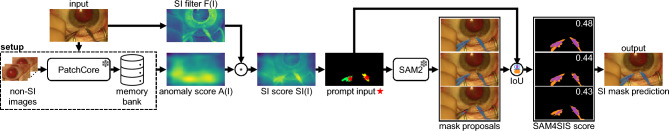
Method overview. In the setup phase, a memory bank is created from images without surgical instrument utilizing the frozen PatchCore encoder. During inference, a PatchCore anomaly score map A(I) is predicted with high values (yellow) for unseen objects. The anomaly map is filtered with the SI filter F(I). From the surgical instrument score map (SI), the input prompt is selected, marked with the red stars. SAM2 proposes three segmentation masks. Based on our SAM4SIS score, the final mask prediction is selected based on the Intersection over Union (IoU) between the mask proposals and SI(I).

Our SIS platform is depicted in Fig. 2. During the setup phase, features are extracted with the frozen PatchCore image encoder and aligned in a memory bank. The inference step comprises five phases: coarse localization of the surgical instrument with PatchCore, refinement of the localization, prompt selection, mask proposal with SAM2 for instrument segmentation, and mask selection.

### Coarse localization with patchCore

We utilize the anomaly detector PatchCore^21^ to obtain an initial coarse localization of the surgical instruments. PatchCore relies on the concept of memory banks that are generated beforehand from nominal patch features extracted from images without anomalies. During inference, patch features from the input image are mapped into the memory bank. An anomaly score is calculated for every pixel based on the distance between the input patch features and their nearest neighbor to the nominal patch features.

**Fig. 3:**
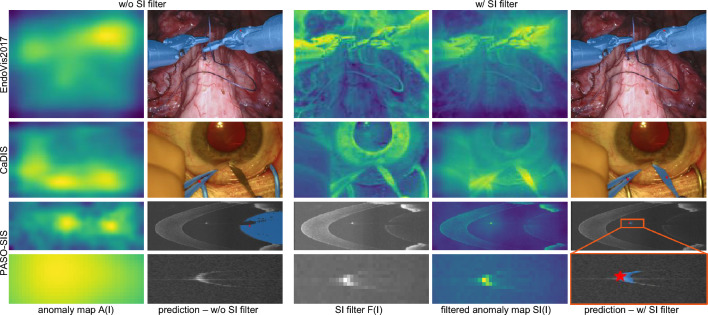
Visualization of the anomaly map A(I), the SI filter F(I), the filtered anomaly map SI(I), and the SAM2 SI mask predictions (blue overlays). The colorscale ranges from blue (low scores) to yellow (high scores). Close-ups of the prediction masks are provided for the PASO-SIS examples. When the SAM2 input prompt is set to the highest score pixel of A(I), one instrument is missing in the CaDIS example. In the PASO-SIS example, the A(I) input prompt does not align with the surgical instrument. Conversely, all surgical instruments are successfully segmented when the input prompt is based on SI(I).

In this work, the set of nominal images are those without surgical instruments present, while surgical instruments are considered anomalies. The memory bank is optimized using a low number of examples with surgical instruments. The resulting anomaly score map $\text {A}(\text {I})$ allows for rough localization of surgical instruments for an image $\text {I}$, but precise segmentation masks are not directly obtained due the coarseness of $\text {A}(\text {I})$, see Fig. 3.

### Refinement of localization: SI score

SAM2 is an LVM for fast and precise promptable segmentation in both images and videos^19^. For an input prompt such as a point or a bounding box referring to the object of interest, it generates a valid segmentation mask. In our approach, we use pixel coordinates as prompt input and automate the prompt selection by utilizing the coarse surgical instrument localization provided by PatchCore.

An apparent candidate for the input prompt is the pixel with the highest anomaly score. However, while the anomaly score is typically high for pixel that belong to surgical instruments and decreases in their environment, the highest anomaly score point may not always overlap exactly with the surgical instrument, see Fig. 3. This is due to the small size of surgical instruments compared to the resolution of $\text {A}(\text {I})$. Additionally, in the presence of unusual structures not represented in the memory bank, areas outside the surgical instrument may receive high anomaly scores as well. Thus, we define a filter $\text {F}(\text {I})$ to generate the surgical instrument score map $\text {SI}(\text {I})$ for an image $\text {I}$ based on its anomaly map $\text {A}(\text {I})$ by pixel-wise multiplication in accordance with $\text {SI}(\text {I}) = \text {A}(\text {I}) \cdot \text {F}(\text {I})$. The filter $\text {F}(\text {I})$ is designed to accommodate different image domains. To cover a wide range of surgical imaging domains, we introduce two filters: one for true color RGB representations and another for intensity-based grayscale representations. The purpose of the filter is the assignment of higher weights to image regions that have a high probability of containing a surgical instrument.

For RGB images, including images of the EndoVis2017 and CaDIS data, the filter $\text {F}_{\text {RGB}}(\text {I})$ is defined as

1 $$\begin{aligned} \text {F}_{\text {RGB}}(\text {I}) = 1 - \frac{\text {I}^{\text {a}^*}/\max ({\text {I}^{\text {a}^*}}) + \text {I}^{\text {V}}/\max ({\text {I}^{\text {V}}}) + \text {I}^{\text {S}^*}/\max ({\text {I}^{\text {S}^*}})}{3}, \end{aligned}$$

based on the observation that surgical instruments are typically (dark) gray and anatomical structures often exhibit a red hue due to abundant blood flow. In Equation 1, $\text {I}^{\text {a}^*}$ is the red-green channel of image $\text {I}$ represented in the CIELAB color space. $\text {I}^{\text {V}}$ and $\text {I}^{\text {S}}$ are the value and saturation channel of the image $\text {I}$ represented in the HSV color space version of $\text {I}$. The first component downweights image parts with high red components independent of the lightning. The second and third component downweigth light and brightly saturated image regions, respectively. The terms are normalized to the maximum value $\max ({\text {I}^{\text {a}^*}})$, $\max ({\text {I}^{\text {V}}})$, and $\max ({\text {I}^{\text {S}}})$ of their corresponding image channel.

The imaging principle for intensity-based modalities like OCT and ultrasound results in shadow regions (i.e., black image regions) below surgical instruments due to their high reflectivity. The shadow regions deviate from the typically noisy OCT signal. Shadows are not represented in the memory bank, resulting them to receive a high anomaly score. To avoid false-positive predictions caused by shadows and ensure that the prediction overlays with an object, we weight the anomaly map $\text {A}(\text {I})$ with the signal intensity by defining the filter for OCT, or more generally, intensity-based imaging modalities, as

2 $$\begin{aligned} \text {F}_{\text {intensity}}(\text {I}) = \text {I}/\max ({\text {I}}), \end{aligned}$$

with the intensity values of $\text {I}$ being normalized between 0 and 1. Figure 3 provides examples of anomaly and surgical instrument score maps.

**Fig. 4:**
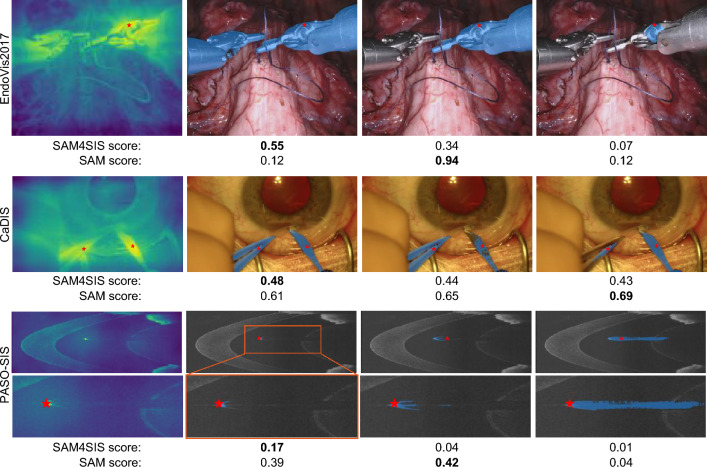
Comparison of SAM and SAM4SIS score-based selection of mask. In both examples, the SAM4SIS score selects the best fitting mask (column 2), while another mask is chosen when utilizing the original SAM score. Close-ups of the prediction masks are provided for the PASO-SIS examples.

### Prompt definition

The pixel with the highest SI score is selected as input prompt for SAM2. For imaging domains with multiple surgical instruments per image (EndoVis2017, CaDIS), we apply spatial clustering of high SI score regions by determining connected components in the binarized SI map. The binarization threshold is determined according to the average size of the surgical instruments relative to the image size. For EndoVis2017, with instruments covering about 35% of the image size, the threshold is set at 0.65. For CaDIS and PASO-SIS, with instruments covering about 15%, the threshold is set at 0.85. For each cluster, the pixel with the highest SI score is selected as input prompt for SAM2, all sharing the same input prompt label.

### Mask proposals with SAM2 and mask selection: SAM4SIS score

To address the ambiguity of prompted object selection, the output of SAM2 consists of three possible segmentation masks $\text {M}_{\text {1}}$, $\text {M}_{\text {2}}$, $\text {M}_{\text {3}}$ with an associated confidence score^19^ (SAM score). Taking the mask with the highest SAM score does not always guarantee a precise segmentation of the surgical instrument, see Fig. 4. To address this, we introduce an own SAM4SIS score $\text {S}^{\text {SAM4SIS}}_i = \frac{|\text {M}_i \cap \text {SI}_{\text {bin}}|}{|\text {M}_i \cup \text {SI}_{\text {bin}}|}$, which is defined as the IoU between the binary surgical instrument mask $\text {SI}_{\text {bin}}$ and the proposed segmentation mask $\text {M}_{i}$ for $i = 1,2,3$. The final segmentation mask $M^*$ is selected as the proposal with the highest SAM4SIS score $\text {M}^* = \arg \max _{i \in \{1, 2, 3\}} \text {S}_i^{\text {SAM4SIS}}$, ensuring that the chosen mask optimally matches the surgical instrument score.

### Code availability

The code used in this study is provided as supplementary material accompanying the manuscript.

## Experimental evaluation

### Data sets

**EndoVIS2017** The EndoVis2017 dataset was published for the Robotic Instrument Segmentation Sub-Challenge^4^ as part of the MICCAI 2017 Endoscopic Vision Challenge. It serves as a benchmark for instrument type segmentation and includes eight videos of robot-assisted surgeries recorded using the da Vinci Xi Surgical System. For videos 1-8, 225 images are designated for training and 75 for testing, while videos 9-10 are left entirely for testing with 300 images each. In accordance with the EndoVis2017 challenge rules, the corresponding training sets are not used when evaluating each of the eight split test sets for videos 1-8. We utilize the binary segmentation masks for evaluation.

Two considerations are necessary for utilizing the dataset for our task: First, according to the challenge definition, the annotation masks only include robotic surgical instruments. Other instruments, such as Drop-in Ultrasound Probes visible in videos 1 and 2, are not considered. The classification of robotic versus non-robotic surgical instruments is beyond the scope of this work, so we exclude videos 1 and 2 from our experiments.

Secondly, the dataset exclusively contains images with surgical instruments. However, the PatchCore setup requires nominal examples without surgical instruments. We address this by extracting 1000 and 300 image patches of size 256 x 256 pixels with and without any instruments, respectively, from the training images using the provided segmentation masks to build the memory bank. For evaluation, we use the full-sized images rather than patches. While this introduces an indirect dependence on surgical instrument (SI) masks, in real surgical scenarios, images without instruments can be acquired before tissue manipulation begins. Therefore, neither the patch extraction step nor SI masks are necessary for deployment.

**Cataract Dataset for Image Segmentation (CaDIS).** CaDIS^5^ is a publicly available dataset for semantic segmentation of cataract surgery videos consisting of 4670 images extracted from 25 videos of the CATARACTS challenge^53^ training set. The videos are acquired with a surgical microscope, providing a top-down view of patient eyes and diverse surgical instruments during cataract surgery. The training set contains 3550 images, the validation set 534 images, and the test set 586 images. All images in the dataset have a resolution of 960$\times$540 pixels. For every pixel, a label from a set of 36 classes covering anatomical structure and surgical instruments is assigned. For our experiments, we convert the multi-class masks to binary background/surgical instrument masks. We evaluate our approach on all images in the test set, i.e. video 2, 12, and 22. In the CaDIS dataset, only few images without surgical instruments are included. To construct the memory bank, we sampled each 500 images with and without surgical instruments from the CATARACTS videos assigned to the train split.

**Porcine Anterior Segment OCT Dataset for Surgical Instrument Segmentation (PASO-SIS).** We employ B-scans from volume scans of ex-vivo porcine eyes acquired with a swept-source 4D OCT system operated at an A-scan rate of 600 kHz^54^. A total of 13 eyes are included in this study, with three scans available for each eye: before, during and after manipulation with a surgical instrument. Surgical instruments typically used in cataract surgery (i.e., cannulas, incision knifes, chopper, phacoemulsifier handpiece) varying in size and shape are utilized by non-surgical experts.

The volumes in the dataset have a resolution of 1408x512x512 pixels, covering a field of view (FOV) of approximately ${5}\,\hbox {mm}$ x ${11.8}\,\hbox {mm}$ x ${11.8}\,\hbox {mm}$. 12 A-scans are repeated in close proximity and averaged for enhanced image quality.

A total of 44,032 B-scans of resolution 512x1408 pixels are extracted from the volumes before and after instrument manipulation. In total, 1020 B-scans are extracted that show the surgical instrument for that we generate binary segmentation masks using Segment Anything Model (SAM) through manual input point prompting and manual correction of proposed masks. These B-scans provide cross-sectional views of surgical instruments along both their longitudinal and transverse axes. To generate the memory bank, we randomly sample 1000 B-scans without and 300 with surgical instruments present. We evaluate our approach on the 1020 available B-scans with surgical instruments. The same 300 images with surgical instruments and their corresponding annotation masks are used to train the supervised baseline model.

### Baseline algorithms

We compare our approach with both an example from an established supervised learning CNN, namely U-Net^51^ and LVM with text prompting, namely GSAM^14^. For a fairer comparison in a limited data scenario, we only use those images with SIs for supervised learning of the U-Net that are provided to SAM4SIS.

**Table 1:** Evaluated text prompts for GSAM. “Surgical instrument” is abbreviated as SI for clarity of the table.

Prompts suggested by Soberanis-Mukul et al.^33^	PASO-SIS-specific prompts
SI.	White line.
SI on the left. SI on the top right corner.	SI in cross-section of the eye.
The grey SI on the skin on the left part of the image. The SI on the right part of the image.	SI in optical coherence tomography B-scan.
The grey SI on the top left. The grey SI on the top right.	Metallic SI in optical coherence tomography B-scan.
The grey SI on the left. The grey SI on the right bottom.	White line in optical coherence tomography B-scan.
The grey SI on the right. The grey SI on the right bottom.	White line in cross-section of the eye.

For GSAM, we utilized the text prompts proposed by Soberanis-Mukul et al.^33^ summarized in Table 1. We investigate two variants: First, we select for each image the prompt that results in the best segmentation mask. Secondly, in contrast to Zhou et al.^31^ and Soberanis-Mukul et al.^33^, we take the same text prompt for all images of each test dataset which avoids manual prompt selection. Furthermore, we test domain-specific text prompts for PASO-SIS, see Table 1.

### Metrics

We use established metrics for pixel-wise binary semantic segmentation including mIoU and mDICE. As the overlap-based metrics mIoU and mDICE have limitations when assessing small structures as surgical instruments in the CaDIS and PASO-SIS data, cf. Maier-Hein et al.^55^, we complete the set of metrics with the uncertainty-aware boundary based normalised surface distance (NSD). The NSD is utilized to quantify the degree of overlap between the two boundaries $S_{A}$ and $S_{B}$ of the predicted and reference segmentation mask *A* and *B* and is defined as

3 $$\begin{aligned} NSD(A,B)^{\tau } = \dfrac{|S_{A} \cap \mathcal {B}_{B,\tau }| + |S_{B} \cup \mathcal {B}_{A,\tau }|}{|S_{A}| + |S_{B}|}. \end{aligned}$$

It incorporates the parameter $\tau$ that determines the permissible difference between the predicted and reference boundaries. This parameter establishes the border regions $\mathcal {B}_{A,\tau }$ and $\mathcal {B}_{B,\tau }$ for each mask that include all pixels located within a specific distance from the boundary. By analysing the inter-rater variability when annotating the surgical instruments, we set this distance to ${10}\,\hbox {pixel}$ for EndoVis2017 and CaDIS and ${50}\,\hbox {mm}$for PASO-SIS, respectively.

We measure the inference time in frames per second (fps) and analyze the setup time which corresponds to the time required for generating the memory bank in the case of our SAM4SIS approach and for retraining the CNN in the case of U-Net.

### Implementation details

**Table 2:** PatchCore parameters.

Parameter	Value
Backbone	densenet121
Layers	denseblock2 & denseblock3
Coreset sampling ratio	0.1
Number of neighbors	9
Image & pixel threshold	F1AdaptiveThreshold

For all experiments, we downscale the images to a resolution of 128$\times$128 pixels for EndoVis2017 and CaDIS and 92x256 for PASO-SIS. We utilize the PatchCore implementation from Akcay et al.^56^. The relevant parameters are summarized in Table 2. We employed the official SAM2 implementation from Ravi et al.^19^ and enabled the multimask_output option, as discussed in the previous chapter. The fullsize images are provided to SAM2. We run the experiments on a workstation equipped with an NVIDIA GeForce RTX 3090 GPU.

## Results

### Effectiveness of prompt selection and SI scoring

The success of the SIS relies heavily on the accurate selection of the prompt input within the surgical instrument contour. Table 3 presents the frequency of correct prompt input selection, meaning that the point prompt is within the reference mask. The prompt input is successfully chosen in 69.34%, 50.43%, and 72.75% of the EndoVis2017, CaDIS, and PASO-SIS test frames, respectively. The utilization of the SI score significantly enhances performance for the CaDIS and PASO-SIS dataset. Without the SI score, only 28.83% and 4.21% of input prompts overlap with the surgical instrument mask for the CaDIS and PASO-SIS data, respectively. For EndoVis2017, the frequency of correct prompt inputs is higher without the SI scoring. However, the resulting mDICE significantly increases when SI scoring is applied.

**Table 3:** Effectiveness of prompt selection and SI scoring mechanism. Comparison of prompt accuracy pAcc and overall SIS performance when input prompt *p* is defined as point with highest value of the anomaly score map $\text {A(I)}$ or surgical instrument score map $\text {SI(I)}$.

	EndoVis2017		CaDIS		PASO-SIS	
	pAcc [%]	mDICE [%] (SD)	pAcc [%]	mNSD [%] (SD)	pAcc [%]	mNSD [%] (SD)
$p = \text {argmax}(\text {A}(\text {I}))$	**75.13**	49.77 (0.43)	28.83	27.95 (0.44)	4.21	16.43 (0.46)
$p = \text {argmax}(\text {SI}(\text {I}))$	69.34	**67.91** (0.39)	**50.46**	**53.42** (0.43)	**72.75**	**72.64** (0.38)

**Table 4:** Effectiveness of SAM2 segmentation and SAM4SIS scoring mechanism.

	EndoVis2017 [%] (SD)		CaDIS [%] (SD)		PASO-SIS [%] (SD)	
	mDICE^*^	mDICE	mNSD^*^	mNSD	mNSD^*^	mNSD
SAM scoring	77.11 (0.12)	62.32 (0.38)	**76.52** (0.11)	53.14 (0.41)	75.03 (0.31)	61.75 (0.34)
SAM4SIS scoring	**82.69** (0.09)	**67.91** (0.39)	73.28 (0.13)	**53.42** (0.43)	**88.17** (0.13)	**72.64** (0.38)

*For successful prompts only

### Effectiveness of segmentation mask proposal and SAM4SIS scoring

We analyze the mDICE and mean normalised surface distance (mNSD) for frames in which the input prompt is successfully selected within the surgical instrument’s reference mask. As shown in Table 4, high segmentation accuracy is achieved for all datasets in case of correct prompt selection, demonstrating the effectiveness of segmentation mask proposal using SAM2 for SIS. We further examine the effectiveness of the proposed SAM4SIS scoring in comparison to the default SAM scoring for selecting one of the three proposed segmentation masks. For EndoVis2017 and PASO-SIS, the SAM4SIS scoring leads to significantly better segmentation masks. For CaDIS, utilizing the original SAM scoring results in superior segmentation masks selection for prompts overlapping with the surgical instrument. However, for all datasets, SAM4SIS scoring selects more suitable masks for incorrect input prompts, leading to an overall slightly better performance with SAM4SIS scoring.

### Comparison to SOTA

**Table 5:** Comparison of methods across different datasets: EndoVis2017, CaDIS, and PASO-SIS.

		Data			Accuracy [%] (SD)			Speed	
Dataset	Method	w/o SI	w/ SI	Masks	mDICE	mIoU	mNSD	Setup time [sec]	fps
EndoVis2017	U-Net^51^	–	191	191	**84.73 (0.02)**	**74.54 (0.03)**	63.16 (0.04)	482	122
	GSAM^*^^14^	–	–	–	67.72 (0.40)	54.27 (0.45)	57.38 (0.42)	–	2
	GSAM^**^^14^	–	–	–	72.84 (0.38)	59.72 (0.43)	**63.47 (0.40)**	*manual prompt selection*	
	SAM4SIS	1457	191	–	67.91 (0.39)	58.56 (0.44)	54.07 (0.41)	14	7
CaDIS	U-Net^51^	–	500	500	38.20 (0.02)	26.10 (0.03)	46.81 (0.02)	351	122
	GSAM^*^^14^	–	–	–	18.58 (0.35)	15.70 (0.37)	28.82 (0.40)	–	2
	GSAM^**^^14^	–	–	–	38.19 (0.42)	31.72 (0.45)	46.12 (0.41)	*manual prompt selection*	
	SAM4SIS	500	500	–	**49.40 (0.41)**	**39.77 (0.44)**	**53.42 (0.43)**	24	7
PASO-SIS	U-Net^51^	–	300	300	17.14 (0.07)	12.38 (0.08)	27.92 (0.06)	316	122
	GSAM^*^^14^	–	–	–	9.68e-5 (0.30)	1.94e-4 (0.30)	1.23e-4 (0.40)	–	2
	GSAM^***^^14^	–	–	–	9.67e-5 (0.30)	1.93e-4 (0.30)	1.21e-4 (0.40)	–	2
	SAM4SIS	1000	300	–	**60.62 (0.39)**	**49.67 (0.42)**	**72.64 (0.38)**	32	7

As summarized in Table 5, the three compared methods, U-Net^51^, GSAM^14^, and SAM4SIS, exhibit distinct strengths and weaknesses. For the EndoVis2017 data, the highest segmentation accuracy and inference speed is achieved with the supervised U-Net model. However, this is countered by the need for ground truth masks for resource-intensive supervised training. For the CaDIS and PASO-SIS datasets, where the variability in surgical instrument appearance is more pronounced, the instruments are smaller, and the distinction from anatomical structures is complicated by reflections and cornea-induced distortions (CaDIS) as well as speckle noise (PASO-SIS), the limited number of training images and annotation masks is insufficient for successful segmentation of the test data. Consequently, the mean Normalized Surface Distance (mNSD) falls below 50% for CaDIS and 30% for PASO-SIS. While GSAM prompted with “surgical instrument” is the most convenient method with no domain data or setup time required, the achieved segmentation accuracy is at the same level for EndoVis2017 and lower for CaDIS compared to our SAM4SIS method. However, our method can only be surpassed with GSAM if an individual prompt is chosen for each image, which incurs costs related to user interaction. For the PASO-SIS data, all tested text prompts, including domain-specific descriptions, failed for selecting the surgical instrument as object of interest. Our SAM4SIS method results in the highest accuracy scores for CaDIS and PASO-SIS. For EndoVis2017, we reach a mDICE of 67.91%. For all datasets, the standard deviation for U-Net results remains below 0.04, while it is significantly higher for GSAM and SAM4SIS, ranging from 0.30 to 0.45.

For SAM4SIS, the inference time is distributed as follows: approximately 90 ms for the PatchCore prediction, 25 ms for SAM2 prediction, and ${27}\,\hbox {mm}$ for prompt and mask selection. With 7 fps, SAM4SIS achieves more than three times higher fps compared to GSAM (7 fps vs 2 fps). Nonetheless, SAM4SIS cannot compete with the 122 fps of the U-Net implementation. It should be noted, however, that the emphasis of this work was not on enhancing the computational speed of any of the utilized models. As a result, all reported number were derived from a basic implementation.

Lastly, a key characteristic of our method is a short setup time of under 32 seconds, which represents one of the most relevant advantages for various downstream tasks and deployment in surgical settings.

**Fig. 5:**
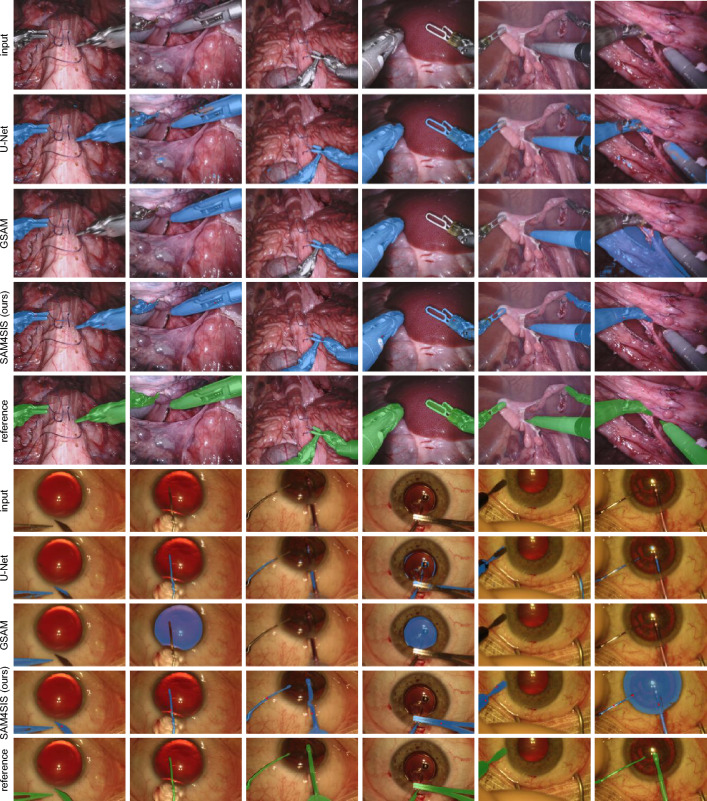
Exemplary segmentation mask results generated with SAM4SIS (ours) and the baseline methods U-Net^51^ and GSAM^14^. The predicted and reference segmentation masks are displayed in blue and green, respectively. The last column presents a failure case for our method.

**Fig. 6:**
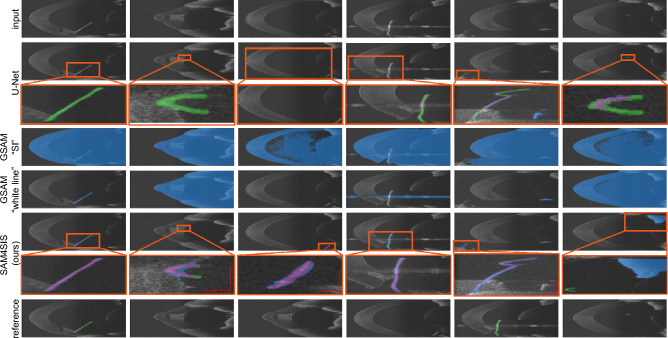
Exemplary segmentation mask results generated with SAM4SIS (ours) and the baseline methods U-Net^51^ and GSAM^14^. The predicted and reference segmentation masks are displayed in blue and green, respectively. In the close-ups for U-Net and SAM4SIS, true-positives, false-positives, and false-negatives are displayed in purple, blue, and green, respectively. The last column presents a failure case for our method. Close-ups of the prediction masks are provided for both U-Net and SAM4SIS predictions.

Figure 5 shows exemplary qualitative results for both tested domains. It demonstrates the effectiveness of detecting SIs of different kinds and orientations. It is notable that our approach is successful despite light reflections or anatomical structures partly occluding the object (CaDIS, column 2 and 4). Furthermore, the whole instrument is segmented even though it is visually separated by its texture (CaDIS, column 5) or geometry (EndoVis2017, column 1-5; CaDIS, column 1; PASO-SIS column 1 and 5). The last column provides a failure case for our approach: a missing mask for the second instrument in the EndoVis2017 domain, a transparent instrument in the CaDIS domain causing the inclusion of the iris and lens area in the segmentation mask, and an anomalous placement of the iris in the PASO-SIS domain, with an appearance not represented in the dataset, thus mistakenly perceived and prompted as an SI. These examples represent typical failure cases of SAM4SIS: incorrect mask selection in situations with ambiguities, despite SAM4SIS scoring, resulting in missing or superfluous segments in the prediction, and incorrect SI prompt selection due to anomalous, non-SI regions in the scene, leading to the wrong object being segmented. In most images, the U-Net masks overlaps at least partly with the instrument. However, the contours of the mask are less precise and smooth. In contrast, GSAM as well as our method, both relying on Segment Anything Model (SAM) or SAM2 as segmentation models, precisely find the contour of segments. However, GSAM selects the wrong object in the majority of cases for the CaDIS and PASO-SIS examples. For PASO-SIS, the text prompt “surgical instrument” is unsuccessful in all shown examples. With the domain-specific descriptive prompt “white line”, the surgical instrument is found in two out of five examples (Fig. 6).

### Domain agnosticism

**Fig. 7:**
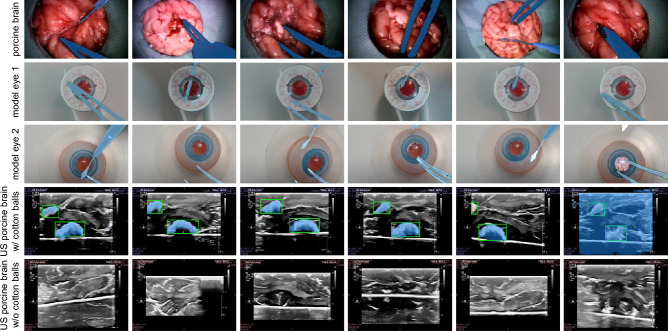
Exemplary SAM4SIS results for SIS in surgical microscope images of ex-vivo porcine eyes, and two types of model eyes, as well as for foreign body (cotton ball) segmentation in ex-vivo porcine brain ultrasound (US) scans are presented. The predicted segmentation masks are displayed in blue and green, respectively. For the US brain scans, the reference bounding box for foreign body detection is displayed in green. For comparison, the last row shows examples of porcine brain US scans without cotton balls present.

To investigate domain agnosticism, we test SAM4SIS on three additional surgical instrument domains, including in-house surgical microscope data from ex-vivo porcine brains, the SimuloRhexis^®^ eye model from sEYEt, LLC, USA (model eye 1), and the VR PS-010 model eye from Phillips Studio, GB (model eye 2). Furthermore, we test our *segment anything new* concept on an additional clinical dataset for the task of foreign body (cotton ball) detection in ex-vivo porcine eye brain ultrasound scans^57^. A distinct feature for the ultrasound dataset is that the images, in addition to the ultrasound B-scans, display patient and system information on a black border around the scan area. This often results in the SAM2 masks segmenting the entire scan from the image border (see Fig. 7, row 4, column 6). To mitigate this, alongside the surgical instrument/cotton ball prompts, a negative prompt was provided to SAM2, corresponding to the lowest SI score in the central area. Figure 7 displays exemplary successful, partially successful, and failure cases, demonstrating that SAM4SIS is capable of addressing the task across various domains.

## Discussion

Our proposed SAM4SIS method achieves accurate segmentation of surgical instruments across the evaluated imaging domains. Among the four phases - instrument localization, input prompt selection, segmentation mask proposal, and segmentation mask selection - input prompt selection has proven to be the most error-prone. However, by effectively combining SAM2 with our SAM4SIS scoring, errors in prompt selection can be mitigated. For instance, if the correct input prompt is chosen for only one of two instruments, the SAM4SIS scoring utilizing the SI score map can still guide the selection of a SAM2 mask that includes both instruments. For the employment of SAM4SIS in diverse surgical domains, neither model (re-)training nor parameter fine-tuning is necessary, apart from the binary selection of the SI filter (true-color RGB filter $\text {F}_{\text {RGB}}(\text {I})$, Equation 1, vs. 1-channel intensity filter $\text {F}_{\text {intensity}}(\text {I})$, Equation 2). Nonetheless, domain-specific optimization of the SI filter or PatchCore parameters can further enhance segmentation performance.

A comparison of segmentation accuracy, along with data and time requirements, underscores the trade-off between the resources needed and the performance achieved by supervised learning models, text-prompted foundation models, and our SAM4SIS model. Our key findings indicate that while SAM4SIS cannot match the performance of supervised learning when sophisticated annotated training data is available (cf. Section 2), it excels when no annotated data is present, including challenging domains such as OCT and US. Additionally, SAM4SIS offers the benefit of a rapid, training-free setup time of 32 seconds or less, which is beneficial for surgical workflow integration. Moreover, promising results on various model eyes demonstrate effectiveness in non-anatomical backgrounds, thus enabling SAM4SIS to support resource-efficient research prototyping in lab setups.

Previous studies such as^33^ have demonstrated the effectiveness of text prompt inputs for the generation of surgical instrument mask annotations in endoscopic videos. However, for automated semantic segmentation we require a consistent text prompt for all images of one dataset, which we were unable to find. Therefore, we conclude that text-prompt based SIS without image-wise user-selection of the text prompt is not yet feasible without adaptations. Furthermore, for the PASO-SIS dataset, where the concept of surgical instruments has a significantly varied appearance compared to e.g., RGB images, we are unsuccessful in localizing the instrument also with describing text prompts like *“white line”*. This demonstrates that the prompt engineering is more challenging for OCT and similar intensity-based cross-sectional imaging domains.

For the EndoVis2017 dataset, the primary failure case for SAM4SIS involves missed instruments when multiple instruments are present. Typically, all instruments are reflected in the anomaly and SI score map, and tests have shown that enhanced results can be achieved by optimizing parameters such as the PatchCore backbone, binarization settings, and the SI filter definition for specific datasets. However, since our focus is on domain agnosticism, we did not adjust the parameters for individual datasets in this work.

Some prominent SIS datasets including the EndoVis2017^4^, do not provide images without surgical instruments. Extracting non-surgical instruments brings the need for SI annotation masks. However, in relevant clinical applications including microsurgery and ophthalmic surgery, frames without surgical instruments can be acquired before the tissue manipulation begins. Furthermore, for most surgical applications, the acquisition of images without SIs is easier to obtain than the acquisition of a dataset with surgical instruments present.

In medical imaging, data often have a three-dimensional or temporal component. In such cases, exploiting the spatial or temporal consistency of the instrument position can enhance the task of SIS. As a future step, we propose incorporating tracking possibilities provided by SAM2 to further reduce outlier predictions and increase inference speed. This approach is particularly advantageous given the high variance and qualitative results, which indicate that SAM4SIS achieves either high segmentation accuracy in successful cases or suboptimal performance in instances of unsuccessful SI prompt selection. By applying consistency assumptions, failure cases might be mitigated.

A further avenue to explore is the extension to SI instance segmentation which is supported by SAM2 by assigning individual prompt labels corresponding to the SI(I) map clusters.

The demonstration of successful employment of SAM4SIS for foreign body detection highlights the potential of our method for segmentation tasks beyond surgical instruments and encourages the transfer of the approach to domains and applications outside the clinical context, where the *segment anything new* idea can be leveraged.

## Supplementary Information

Supplementary Information 1.

## Data Availability

This study uses four publicly available datasets: The EndoVis2017 dataset, which can be requested via https://dx.doi.org/10.21227/ac97-8m18, the CaDIS dataset, accessible at https://ieee-dataport.org/open-access/cataracts, our PASO-SIS dataset, accessible at https://doi.org/10.7910/DVN/HUCGAE, and the ultrasound brain dataset for foreign body detection in neurosurgery, accessible at https://dx.doi.org/10.21227/v4y4-k985. The in-house surgical microscopic images from porcine brains and model eyes can be requested via the corresponding author.
